# Hepatic and Vascular Vitamin K Status in Patients with High Cardiovascular Risk

**DOI:** 10.3390/nu13103490

**Published:** 2021-10-01

**Authors:** Nikolas Rapp, Vincent M. Brandenburg, Nadine Kaesler, Stephan J. L. Bakker, Robert Stöhr, Alexander Schuh, Pieter Evenepoel, Leon J. Schurgers

**Affiliations:** 1 Department of Biochemistry, Cardiovascular Research Institute Maastricht (CARIM), Maastricht University, 6200 MD Maastricht, The Netherlands; n.rapp@maastrichtuniversity.nl; 2 Department of Cardiology, Rhein-Maas-Klinikum Würselen, 52146 Würselen, Germany; vincent.brandenburg@post.rwth-aachen.de; 3 Department of Nephrology and Clinical Immunology, RWTH Aachen University Hospital, 52074 Aachen, Germany; nkaesler@ukaachen.de; 4 Institute of Experimental Medicine and Systems Biology, RWTH Aachen University Hospital, 52074 Aachen, Germany; 5 Division of Nephrology, Department of Internal Medicine, University Medical Center Groningen, University of Groningen, 9713 GZ Groningen, The Netherlands; s.j.l.bakker@umcg.nl; 6 Department of Cardiology, University Hospital of the RWTH Aachen, 52074 Aachen, Germany; rstoehr@ukaachen.de (R.S.); aschuh@ukaachen.de (A.S.); 7 Department of Microbiology, Immunology and Transplantation, Laboratory of Nephrology, KU Leuven and University Hospitals Leuven, 3000 Leuven, Belgium; pieter.evenepoel@uzleuven.be

**Keywords:** vitamin K, vitamin K dependent proteins, cardiovascular disease, dp-ucMGP, PIVKA-II, chronic kidney disease, calciphylaxis

## Abstract

Vitamin K dependent proteins (VKDP), such as hepatic coagulation factors and vascular matrix Gla protein (MGP), play key roles in maintaining physiological functions. Vitamin K deficiency results in inactive VKDP and is strongly linked to vascular calcification (VC), one of the major risk factors for cardiovascular morbidity and mortality. In this study we investigated how two vitamin K surrogate markers, dephosphorylated-undercarboxylated MGP (dp-ucMGP) and protein induced by vitamin K absence II (PIVKA-II), reflect vitamin K status in patients on hemodialysis or with calcific uremic arteriolopathy (CUA) and patients with atrial fibrillation or aortic valve stenosis. Through inter- and intra-cohort comparisons, we assessed the influence of vitamin K antagonist (VKA) use, vitamin K supplementation and disease etiology on vitamin K status, as well as the correlation between both markers. Overall, VKA therapy was associated with 8.5-fold higher PIVKA-II (0.25 to 2.03 AU/mL) and 3-fold higher dp-ucMGP (843 to 2642 pM) levels. In the absence of VKA use, non-renal patients with established VC have dp-ucMGP levels similar to controls (460 vs. 380 pM), while in HD and CUA patients, levels were strongly elevated (977 pM). Vitamin K supplementation significantly reduced dp-ucMGP levels within 12 months (440 to 221 pM). Overall, PIVKA-II and dp-ucMGP showed only weak correlation (r^2^ ≤ 0.26) and distinct distribution pattern in renal and non-renal patients. In conclusion, VKA use exacerbated vitamin K deficiency across all etiologies, while vitamin K supplementation resulted in a vascular VKDP status better than that of the general population. Weak correlation of vitamin K biomarkers calls for thoughtful selection lead by the research question. Vitamin K status in non-renal deficient patients was not anomalous and may question the role of vitamin K deficiency in the pathogenesis of VC in these patients.

## 1. Introduction

The unequivocal role of vitamin K is to mediate the posttranslational gamma-glutamyl carboxylation of specific, protein bound glutamate (Glu) residues into gamma-carboxyglutamate (Gla) residues. Hence, proteins carrying these residues are called vitamin K-dependent proteins (VKDP). VKDP play a well-known major role in the coagulation system (factors II, VII, IX and X). Another prototypic VKDP is MGP. MGP represents a major, local anti-calcification factor in the vascular wall, and thus protects against vascular calcification (VC) [1]. VKDP rely upon sufficient vitamin K for full biological activity [2,3,4]. The undercarboxylated fraction of VKDP can be specifically measured in the blood, which, as a marker, reflects functional vitamin K status more robustly than direct vitamin K measurements [5]. However, measuring a vitamin K surrogate marker cannot determine the status of different vitamin K species, such as phylloquinone and menaquinones, because both subtypes can be used as cofactor and are bioactive, and because after absorption, vitamin K can be converted into menaquinone-4 [6]. While menaquinones are the prevalent active form in extra hepatic tissue, and hence the vessel wall [7], there is currently no data available that points to any of the surrogate markers showing correlation with a specific vitamin K subtype. Thus, using vitamin K-dependent proteins as surrogate markers reflects all vitamin K forms. Synthesized in different cell types within the body, the undercarboxylated fractions of some VKPD have been linked to the vitamin K status of certain tissues. dp-ucMGP has been described as a parameter to assess vascular vitamin K status, while (PIVKA-II) reflects hepatic vitamin K status [5]. Increasing the supplementation with vitamin K substantially increases the carboxylation status of these marker proteins [8,9], while application of vitamin K antagonists (VKA) or oral anticoagulant therapy (OAT) interferes with the carboxylation process of Gla proteins [10]. Thus dp-ucMGP and PIVKA-II can be used to assess the efficacy of vitamin K supplementation or the impact of VKA therapy [2,11]. 

We investigated plasma samples from patients with four different disease backgrounds. Chronic kidney disease (CKD) patients on hemodialysis (HD), CKD patients undergoing HD with calcific uremic arteriolopathy (CUA), patients with atrial fibrillation (AF) and patients with aortic valve calcification (AVC). All groups have either established or are at increased risk of cardiovascular (CV) disease, and have confirmed or are at high risk of VC. Considering the increasingly acknowledged role of vitamin K in CV health, the aim of the present study was threefold. Firstly, we sought to determine whether vitamin K levels differ between patients representing a wide range of CV disease burden and CKD by the means of dp-ucMGP and PIVKA-II, and how each group compares to the general population. Secondly, we investigated if both surrogate markers reflect the vitamin K status in a similar manner. Thirdly, we studied how VKA treatment or vitamin K supplementation affected vitamin K status in our cohorts.

## 2. Materials and Methods

### 2.1. Design and Patients 

We performed a post hoc analysis on data collected in the frame of 4 different cohort studies including: HD patients scheduled for kidney transplantation (NCT01886950, Leuven, Belgium); HD patients with CUA (German calciphylaxis registry, Aachen [12]); patients with aortic valve calcification enrolled in a randomized controlled trial to placebo versus vitamin K1 supplementation (“Vitamin K Supplementation for Inhibition of the Progress in Aortic Valve Calcification”, NCT00785109 [8]); and patients with AF randomized to rivaroxaban versus phenprocoumon (“Rivaroxaban Compared to Vitamin K Antagonist Upon Development of Cardiovascular Calcification”, NCT02066662, Aachen [2]).

### 2.2. Hemodialysis Patients (HD)

Forty HD patients, of whom 20 were treated with a VKA, were recruited from an ongoing, prospective observational study investigating outcomes after kidney transplantation. The study has been registered as a clinical trial with the unique identifier NCT01886950. All patients eligible for renal transplantation were included, excluding only bisphosphonate users. Blood samples were collected at the time of the call for transplantation (random, non-fasting) and stored at −80 °C until use. Demographic characteristics and data on immunosuppressive and mineral metabolism therapy were retrieved from electronic files

### 2.3. Hemodialysis Patients with Calcific Uremic Arteriolopathy (CUA)

Thirty-two CUA patients were randomly selected from the German calciphylaxis registry. Detailed results of the entire registry cohort were previously published [12]. In brief, the German calciphylaxis registry is a prospective, nation-wide, internet-based registry in which treating physicians can notify cases of CUA. Confirmation of the diagnosis is based on a plausibility check of the incoming data about medical history, clinical aspects and picture documentation when available. A plausibility check in cases of doubt includes verification discussion between both parties. The registry team at the Aachen University Hospital requested full blood, plasma and serum sampling of the patients according to standard procedures, and asked for immediate freezing at the peripheral study site. Long-term storage was done at −80 °C immediately after arrival at the central biobank at the RWTH Aachen University Hospital. We analyzed stored blood samples of patients on long-term HD prior to the diagnosis of CUA. Patients were divided into two groups according to VKA treatment longer than 6 months prior to the diagnosis (*n* = 14) or no VKA prior to the diagnosis of CUA (*n* = 18). 

### 2.4. Patients with Aortic Valve Calcification (AVC)

We analyzed stored blood samples of a previously published, randomized, controlled, open-label study about the influence of 2 mg vitamin K1 (daily, orally administered phytomenadion) application per day over 12 months compared to placebo in AVC patients. Data of an intention-to-treat cohort were previously published and the study has been registered as a clinical trial with the unique identifier NCT00785109 [8]. Exclusion criteria for this mono-center, open, controlled, randomized phase I study were, amongst others, chronic kidney disease, recent additional vitamin K intake, oral anticoagulation with vitamin K antagonists, venous thrombosis or embolization of lung arteria. Presence of aortic valve calcification was confirmed via echocardiography. For the present analysis, we included all patients for whom stored blood samples at baseline and at 12 months were available. These patients had no or only mild chronic kidney disease (Table 1).

### 2.5. Patients with Atrial Fibrillation (AF)

We randomly selected 30 patients from a randomized, prospective interventional study in which the influence of VKA upon coronary artery calcification in patients with AF or deep vein thrombosis/pulmonary embolism is evaluated compared to rivaroxaban, registered as a clinical trial with the unique identifier NCT02066662. Main inclusion criteria were existent coronary or valvular calcification, or both, an Agatston score > 50 in at least one location and need for long term anti-coagulant therapy. Relevant exclusion criteria were liver disease with coagulopathy or other bleeding disorders including cirrhotic patients with Child–Pugh B and C, clinically significant active bleeding and acute gastrointestinal disease. Coronary artery calcification and AVC were assessed by multi-slice spiral computed tomography scanning. Blood samples of patients after 12 months of randomized treatment were selected. These patients had no or only mild chronic kidney disease (Table 1). 

### 2.6. Individuals from the General Population (Control)

The present study included individuals of the LifeLines-MINUTHE (Micronutrients and Health disparities in Elderly) subcohort of the LifeLines Cohort Study. This subcohort consists of 1605 individuals aged between 60 and 75 years with available plasma, serum and 24-h urine samples from the biobank of the LifeLines cohort. Fasting blood samples are processed on the day of collection and stored at −80 °C. The 1605 individuals were comprised of 400 men and 403 women with low socioeconomic status (SES) and 402 men and 400 women with high SES. Since education is more differentiating than income in the Dutch population, the classification of SES was based on educational status. Low SES was defined as having never been to school or having been to elementary school only, or having completed lower vocational or secondary schooling; high SES was defined as having completed higher vocational schooling or education [13,14,15]. Of this subpopulation, 65 individuals, age matched to disease cohorts, were randomly selected for the present study.

### 2.7. Biochemistry

Blood samples were collected in either potassium-ethylenediaminetetraacetic acid (EDTA) or serum vacutainers, immediately centrifuged and stored in aliquots at −80 °C. Circulating dp-ucMGP levels were determined in EDTA plasma using the commercially available IVD CE marked chemiluminescent InaKtif MGP assay on the IDS-iSYS system (IDS, Boldon, UK). A total of 50 μL of patient sample or calibrators are incubated with magnetic particles coated with murine monoclonal dpMGP antibody, an acridinium labelled murine monoclonal ucMGP antibody and assay buffer. The magnetic particles are captured using a magnet and a wash step is performed to remove any unbound analyte. Trigger reagents are added, and the resulting light emitted by the acridinium label is directly proportional to the concentration of dp-ucMGP in the sample. The within-run and total precision of this assay were 0.8–6.2% and 3.0–8.2%, respectively. The assay measuring range is between 300–12,000 pmol/L and was found to be linear up to 11,651 pmol/L. dp-ucMGP values below 300 pmol/L are considered to be in the normal healthy range. Assays were performed in a single run by Coagulation Profile BV, Maastricht, the Netherlands. 

Circulating PIVKA-II (ucFII) levels were measured using a conformation-specific monoclonal antibody in an ELISA-based assay [16]. Results are expressed as arbitrary units per liter (AU/l) because in states of vitamin K deficiency, circulating ucFII may comprise multiple forms of partially carboxylated FII and neither their relative abundance in serum nor their relative affinity for the antibody is known. Using electrophoretic techniques, 1 AU is equivalent to 1 μg of purified ucFII [16]. The detection limit was 0.15 AU/mL ucFII in serum. Other routine mineral metabolism parameters were extracted from the (electronic) patient files. Parathyroid hormone levels are reported as times upper normal limit (UNL) to account for inter assay variability. 

### 2.8. Statistical Analyses

**Figure 1:**
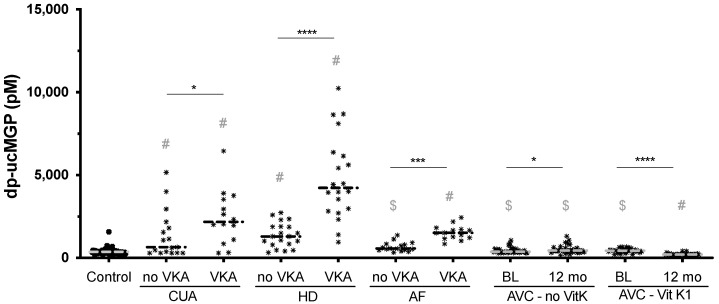
dp-ucMGP measurements of all disease cohorts and a control group. VKA use exacerbated dp-ucMGP status in all cohorts, while vitamin K supplementation ameliorated the dp-ucMGP status beyond healthy controls. Compared to controls, all HD and CUA groups had significantly increased dp-ucMGP levels, while in the AF cohort only the VKA group was increased. Dash indicates the median. Significance levels: * = *p* ≤ 0.05; *** = *p* ≤ 0.001; **** = *p* ≤ 0.0001. # and $ indicate the comparison between each disease group and the control group. # indicates a significance level of *p* ≤ 0.0001, and $ indicates no significant difference between the respective group and the control group.

Prior to analysis, data was square root transformed. Plasma dp-ucMGP concentration as well as PIVKA-II plasma levels are compared using ordinary one-way ANOVA with the Tukey post hoc test for multiple comparisons (no VKA and VKA groups of the CUA, HD and AF cohorts and comparison between all diseased groups and the control group). The Wilcoxon matched-pairs signed-rank test has been used to compare the paired AVC study groups. The Kruskal–Wallis test, followed by Dunn’s post hoc correction for multiple comparisons, was used to compare the AVC groups to the control group. Ordinary one-way ANOVA has been used to analyze differences in the clinical characteristics for continuous variables and the Chi-Square test for proportional data. To assess the correlation between dp-ucMGP and PIVKA-II calculation has been performed as Pearson correlation. Significance levels are displayed as: Ns = *p* > 0.05; * = *p* ≤ 0.05; ** = *p* ≤ 0.01; *** = *p* ≤ 0.001; **** = *p* ≤ 0.0001. In Figure 1, # also indicates a significance level of *p* ≤ 0.0001 and $ indicates no significant difference.

## 3. Results

In total, 40 chronic HD patients (20 VKA/20 no VKA), 32 CUA patients (14 VKA/18 no VKA), 30 AF patients (14 VKA/16 no VKA) and 66 AVC patients (34 no vitamin K/32 vitamin K) were included in this study. The patients’ clinical characteristics are shown in Table 1. There was a significant difference in age between the cohorts (*p* ≤ 0.0001), with the HD patients being younger. Furthermore, PTH (*p* ≤ 0.0001), serum calcium (*p* ≤ 0.01) and serum phosphate (*p* ≤ 0.0001) levels showed significant differences between the HD and CUA group (Table 1). 

**Figure 2:**
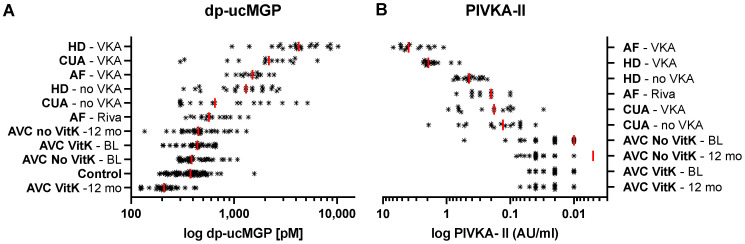
dp-ucMGP and PIVKA-II measurements of all cohorts sorted by median values from high (top) to low (bottom). Red line indicates the median. **A**: VKA treated patients showed higher dp-ucMGP compared to untreated patients of the same cohort. AVC dp-ucMGP levels were lowest with Vitamin K supplemented patients below the control group. **B**: PIVKA-II ranks differ from the dp-ucMGP ranks. AF and HD patients showed highest PIVKA-II levels, followed by CUA and AVC.

To gain an overview of the vitamin K status, all groups were separated by PIVKA-II and dp-ucMGP measurements and ranked by their respective median values. Among all groups, dp-ucMGP levels are higher in patients using VKA, with HD and CUA patients showing higher levels than AF patients. AVC patients supplemented with vitamin K1 showed the lowest dp-ucMGP plasma levels (Figure 2A). For PIVKA-II, the rank was different. AF–VKA patients showed higher PIVKA-II levels. CUA patients have lower PIVKA-II plasma levels than AF and HD patients. AVC patients supplemented with vitamin K1 showed the lowest PIVKA-II levels. (Figure 2B).

Compared to the control group, HD and CUA groups show substantially higher dp-ucMGP levels regardless of VKA use, indicating a strong functional vitamin K deficiency in the dialysis patients. AF patients exhibited values slightly higher than controls, but levels were much higher with VKA use. None of the AVC groups at baseline did show dp-ucMGP values significantly different from the control group. Hence, AF and AVC patients might not by default be characterized by insufficient vitamin K status (Supplemental Table S2).

**Figure 3:**
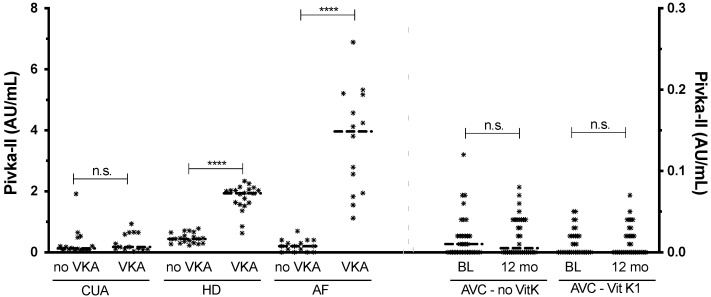
PIVKA-II measurements of all cohorts. HD and AF patients had higher PIVKA-II levels with VKA use. CUA and AVC cohorts did not change with VKA or vitamin K supplementation, respectively. CUA, HD and AF values correspond to the left Y-axis, and AVC values correspond to the right Y-axis. The dash indicates the median. Significance levels: n.s. = *p* > 0.05; **** = *p* ≤ 0.0001.

Next, we compared VKA users against non-users of each disease. dp-ucMGP levels of VKA treated patients were significantly higher for the CUA (*p* ≤ 0.0001, 1.8-fold higher), HD (*p* ≤ 0.0001; 3.5-fold higher) and AF (*p* ≤ 0.001, 2.3-fold higher) cohorts. PIVKA-II levels of VKA treated patients were significantly higher for the HD (*p* ≤ 0.0001, 3.9-fold higher) and AF (*p* ≤ 0.0001; 16.6-fold higher) cohorts only. In the AVC cohort, patients not receiving vitamin K1 supplements did show significantly higher dp-ucMGP levels after 12 month (*p* ≤ 0.05), while in patients receiving the supplements, levels were significantly lower (*p* ≤ 0.0001). PIVKA-II levels, conversely, remained stable in either AVC group. (Figure 2 and Figure 3, Supplemental Table S1).

**Figure 4:**
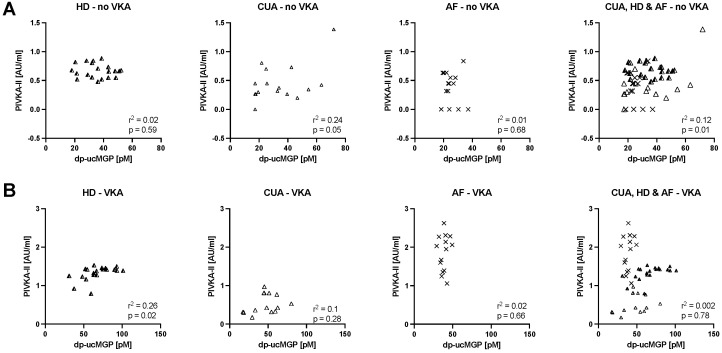
**Weak** correlations between PIVKA-II and dp-ucMGP of all patients of the CUA, HD and AF cohorts, separated into panel **A**) without VKA treatment (r^2^ ≤ 0.24) and panel **B**) with VKA treatment (r^2^ ≤ 0.26). Symbols in the right most graph correspond to the respective patient group.

Furthermore, the vitamin K status of CUA and HD patients was compared. Overall dp-ucMGP levels were similar, with levels being modestly higher only in HD patients treated with VKA. HD patients also showed over all higher PIVKA-II concentrations, an observation that was not made in patients not receiving VKA (Supplemental Figure S1). Finally, we investigated the correlation between dp-ucMGP and PIVKA-II levels in the various cohorts. Correlation between both markers was generally very weak (r^2^ ≤ 0.26, Figure 4A). HD, CUA and AF patients showed a distinct distribution pattern of PIVKA-II and dp-ucMGP. HD and CUA cohorts revealed a pattern skewed towards higher dp-ucMGP, while the AF cohort showed a pattern skewed towards higher PIKVA-II, especially in those treated with VKA (Figure 4B). Moreover, pooling the measurements from all patients of the AF, CUA and HD cohorts did not reveal a good correlation between dp-ucMGP and PIVKA-II either on or off VKA.

## 4. Discussion

Our study compares, for the first time, indicators of vitamin K status in various diseases known for their cardiovascular disease burden. Moreover, assessing the influence of either VKA treatment or vitamin K substitution upon markers of vitamin K status is a unique feature of the present study. Although a detailed quantification of the amount of cardiovascular calcification was not available in these patients (e.g., via autopsy or CT scanning), our data nevertheless adds substantially to our understanding of how vitamin K status, antagonist therapy and supplementation might associate with CV calcification in humans.

HD patients are known to have increased risk for vascular calcifications and cardiovascular mortality [17]. Furthermore, the plasma vitamin K status is known to deteriorate with CKD progression [18] and, reflected by dp-ucMGP, independently predicts CV calcification burden [19]. Vascular calcifications are a hallmark of CUA, but risk factors for development are still ill defined. Vitamin K status and vitamin K antagonist treatment as anti-coagulation therapy have been suspected to increase the odds for the development of CUA, but study results have been inconsistent [20]. 

Somewhat surprising, the vitamin K status in dialysis patients presenting with CUA was not worse compared to counterparts free of the devastating complication. This observation conflicts with a study demonstrating a significantly reduced relative cMGP concentration and a higher prevalence of vitamin K deficiency reflected by PIVKA-II in CUA patients [21]. While in the present study vitamin K deficiency is not more severe in CUA cases than HD patients, our findings do not oppose a contribution of VKA use to the development of CUA per se. The exact mechanism leading to calcific and thrombotic events in subcutaneous arterioles is still unknown. Next to the effect of inhibiting MGP carboxylation, VKAs have additional detrimental effects on human endothelial cells (hEC) and vascular smooth muscle cells (hVSMC) that could fuel CUA development. VKA treatment decreases the carboxylation and secretion of protein S, a coagulation inhibitor also synthesised by hVSMC and hEC [22,23]. Furthermore, VKAs induce hVSMC calcification via endoplasmic reticulum stress, a process independent of Gla protein activation [24]. The precise role of how the coagulation system is involved in CUA development is, to date, unclear. However, occluding thrombus formations in the dermal arterioles with subsequent hypoxia and skin necrosis are events known to contribute to CUA pathology [25]. Patients with CUA show a high prevalence of thrombophilia, underscoring the potential importance of congenital and acquired thrombotic propensity, potentially contributing to the pathogenesis of this disease [26,27]. Therefore, an in-depth assessment of the coagulation system in patients with CUA, preferably prior to initiation of the disease, could potentially yield new treatment strategies for this, to date, difficult to handle complication. 

AF patients included in this study were previously diagnosed with either coronary or valvular calcification and were enrolled in an ongoing trial assessing the influence of VKA treatment on calcification progression (NCT02066662). Remarkably, AF patients with calcifications do not exhibit a strong vitamin K deficiency. However, VKA therapy was associated with considerably higher PIVKA-II levels while differences in dp-ucMGP were much lower. This response clearly opposes what was observed in dialysis patients, in whom VKA therapy predominantly resulted in higher dp-ucMGP levels. This observation fuels the hypothesis that especially vascular vitamin K stores are critically depleted in dialysis patients. The discrepancy might also be partially related to differences in the half-life of MGP (<15 h) [28] and PIVKA-II (62h) [29] and the filtration rate of both proteins. MGP can be filtered by either the kidneys or a dialysis membrane, whereas PIVKA-II is mostly retained [30,31]. Thus, kidney function might be related to circulating plasma levels. However, in the vessel wall of CKD patients MGP is expressed 5-fold higher compared to non-CKD controls, and since vitamin K supplementation decreases dp-ucMGP levels in CKD patients, it indicates a filtration independent effect [32,33]. Recent studies found that vitamin K supplementation in CKD does improve the carboxylation status of VKDP, but without slowing VC progression [33,34]. Vascular calcification in CKD has multiple drivers, including vitamin K deficiency. However, other factors like phosphate disbalance [35] and uremic toxins [36] also contribute, and thus mono therapy with vitamin K might not be sufficient to slow the progression of VC. The CKD uniqueness is supported by the contrasting results of the AVC cohort, as well as other non-CDK cohorts, where vitamin K supplementation was capable of lowering dp-ucMGP levels even below levels observed in healthy controls and consequently attenuated progression of AVC [8,37]. Overall, research coverage on the effect of vitamin K supplementation in VC remains unsatisfactory, and several other studies are currently under way to build a stronger data foundation [38,39].

In the present study, the correlation between PIVKA-II and dp-ucMGP was weak. This might partially be explained by a limited assay sensitivity for PIVKA-II and the therefor reduced precision while measuring lower concentrations. However, other publications investigating CKD and non-CKD samples also reported very weak [40,41] or weak [42] to moderate [11] correlation, while one time a strong [43] correlation between both markers was reported. Moreover, the uncarboxylated fraction of other VKDP such as osteocalcin and the Growth-arrest-specific protein-6 correlate poorly with PIVKA-II and dp-ucMGP [40,44]. This might potentially indicate that VKDP may be subject to relevant regulatory influences other than vitamin K status, and isolated interpretation of individual markers might be difficult. 

The results of the present study should be interpreted considering its limitations. Data on indication, duration, dose and target INR of VKA treated patients are not available. Information on phosphate binder treatment and residual renal function are equally lacking. There are, however, no reasons to assume a systematic bias by these (potential) confounders. 

## 5. Conclusions

The observation of normal vitamin K status in non-renal patients with established VC (AVC and AF) questions the role of vitamin K in the pathogenesis of VC in these patients. PIVKA-II levels and dp-ucMGP show only weak correlations at best, supporting the thesis that these markers most probably represent different functional vitamin K stores, which, moreover, are poorly related. VKA therapy was strongly associated with higher dp-ucMGP levels in all cohorts, but without VKA elevated in HD patients only. Vascular vitamin K stores thus are especially compromised in dialysis, rendering these patients susceptible to accelerated VC. Finally, vitamin K status was not inferior in CUA patients compared to HD patients free of this complication. Yet, VKA use might still be a driver of CUA development by influencing coagulation and VC via vitamin K independent mechanisms. Given the design of our study and inherent limitations, our data should be considered hypothesis-generating, but also call for additional studies.

## Figures and Tables

**Table 1:** Clinical characteristics.

	Hemodialysis		Calcific Uremic Arteriolopathy		Atrial Fibrillation		Aortic Valve Calcification		
	VKA No	VKA Yes	VKA No	VKA Yes	VKA No	VKA Yes	Vitamin K No	Vitamin K Yes	*p* Value (ANOVA)
Age in years	57 ± 13	57 ± 13	69 ± 17	72 ± 14	71 ± 11	69 ± 9	69 ± 8	69 ± 10	≤0.0001
male %	85	70	69	63	65	67	73	79	≤0.01
PTH × UNL	5.57 ± 2.22	3.14 ± 2.22	2.40 ± 2.05	2.08 ± 3.23	nm	nm	nm	nm	≤0.0001
Calcium mmol/L	2.3 ± 0.1	2.3 ± 0.1	2.2 ± 0.1	2.2 ± 0.2	2.4 ± 0.1	2.3 ± 0.1	2.3 ± 0.2	2.4 ± 0.2	≤0.01
Phosphate mmol/L	1.52 ± 0.61	1.42 ± 0.52	0.5 ± 0,13	0.53 ± 0.14	nm	nm	nm	nm	≤0.0001
month on dialysis	43 ± 32	49 ± 46	46 ± 17	30 ± 21	not applicable	not applicable	not applicable	not applicable	0.39
hypertension %	75	60	91	89	74	78	78	71	≤0.0001
Diabetes mellitus %	20	35	56	44	23	25	29	36	≤0.0001
eGFR mL/min/1.73^2^	all dialysis	all dialysis	all dialysis	all dialysis	63 ± 12	71 ± 15	71 ± 14	69 ± 18	0.36

Compared to the control group, HD and CUA groups show substantially higher dp-ucMGP levels, regardless of VKA use, indicating a strong functional vitamin K deficiency in the dialysis patients. AF patients exhibited values slightly higher than controls, but levels were much higher with VKA use. None of the AVC groups did show dp-ucMGP values significantly different from the control group. Hence, AF and AVC patients might not by default be characterized by insufficient vitamin K status (Supplemental Table S1).

## Data Availability

The data presented in this study are available on request from the corresponding author.

## Supplementary Materials

The following are available online at https://www.mdpi.com/article/10.3390/nu13103490/s1, Supplemental Table S1: dp-ucMGP and PIVKA-II measurements for each cohort, Supplemental Table S2: dp-ucMGP measurements: patients vs. healthy controls, and Supplemental Figure S1: dp-ucMGP and PIVKA-II levels of CUA and HD cohorts.
