# Supplementary material for: Hepatic and Vascular Vitamin K Status in Patients with High Cardiovascular Risk
Source: Nutrients. 2021 Oct 1;13(10):3490. doi: 10.3390/nu13103490 (PMC8539359; doi:10.3390/nu13103490)
Supplement: Supplementary file 1 [file nutrients-13-03490-s001.zip › nutrients-1346107-SI.pdf]

Table S1. dp-ucMGP and PIVKA-II measurements for per cohort.

CUA	no VKA	VKA	Foldchange	p value$^{\$ / \#}$
dp-ucMGP [pM]	1384 ± 1446	2443 ± 1649	1.8	0.001
PIVKA-II [AU/ml]	0.2 ± 0.19	0.34 ± 0.3	1.7	0.99
**HD**	**no VKA**	**VKA**		
dp-ucMGP [pM]	1385 ± 7478	4844 ± 2551	3.5	<0.0001
PIVKA-II [AU/ml]	0.46 ± 0.17	1.79 ± 0.44	3.9	<0.0001
**AF**	**Rivaroxaban**	**VKA**		
dp-ucMGP [pM]	671 ± 286	1535 ± 434	2.3	0.002
PIVKA-II [AU/ml]	0.22 ± 0.2	3.65 ± 1,72	16.6	<0.0001
**AVC**				
No vitamin K	**Baseline**	**12 months**		
dp-ucMGP [pM]	463 ± 189	508 ± 251	1.1	0.02
PIVKA-II [AU/ml]	0.02 ± 0.03	0.02 ± 0.02	1	0.78
Vitamin K	**Baseline**	**12 months**		
dp-ucMGP [pM]	437 ± 221	221 ± 72	0.5	<0.0001
PIVKA-II [AU/ml]	0.01 ± 0.02	0.01 ± 0.02	1	0.68

Data are presented as mean ± SD.

Table S2. dp-ucMGP measurements: patients vs. healthy controls.

Group	dp-ucMGP [pM]	p value: control - disease
**Control**	399 ± 190	Does not apply
**CUA**		
**VKA**	2443 ± 1649	<0.0001
**no VKA**	1384 ± 1446	<0.0001
**HD**		
**VKA**	4844 ± 2551	<0.0001
**no VKA**	1385 ± 7478	<0.0001
**AF**		
**VKA**	1535 ± 434	<0.0001
**Rivaroxaban**	671 ± 286	0.36
**AVC**		
No Vitamin K		
**BL**	463 ± 189	0.42
**12 months**	508 ± 251	0.09
Vitamin K		
**BL**	437 ± 221	0.2
**12 months**	221 ± 72	<0.0001

Data are presented as mean ± SD. Significance levels: n.s. =P > 0.05; * = P ≤ 0.05; ** = P ≤ 0.01; *** = P ≤ 0.001; **** = P ≤ 0.0001.

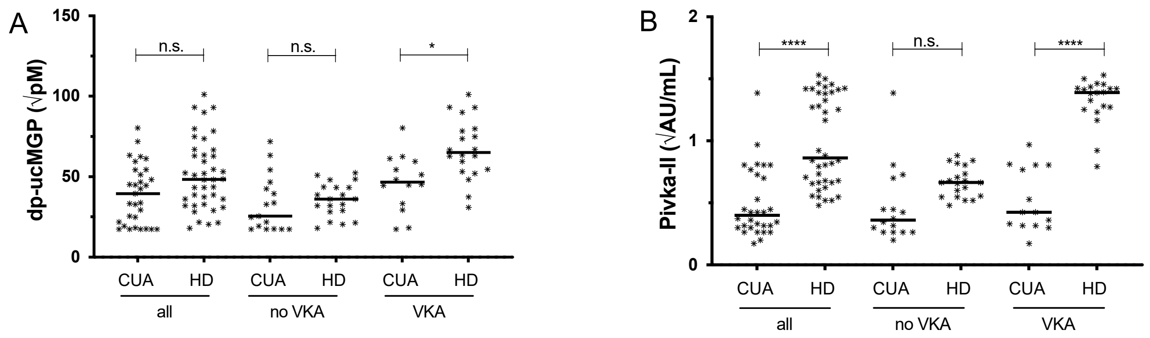

Figure S1. HD patients tended to have a worse vitamin K status than CUA patients as reflected by dp-ucMGP and PIVKA-II. A: dp-ucMGP levels were higher in HD only in the VKA treated group. B: PIVKA levels were higher overall, and in the VKA treated group but not in the non-VKA group.
